# Psychometric properties of the social isolation and social network scale in community-dwelling older adults: Construct validity, reliability, and sensitivity

**DOI:** 10.1371/journal.pone.0338522

**Published:** 2025-12-11

**Authors:** Kang-Hyun Park, Seong-Kyu Ha

**Affiliations:** 1 Department of Occupational Therapy, Baekseok University, Cheonan-Si, South Korea; 2 Department of Occupational Therapy, College of Health and Biology, Semyung University, Jecheon-Si, South Korea; Saint Francis University, HONG KONG

## Abstract

**Background:**

Social isolation is associated with numerous negative health outcomes in older adults. Despite growing concerns about social disconnection, there remains a need for psychometrically sound instruments that comprehensively assess both social isolation and social networks, particularly in culturally diverse contexts.

**Objective:**

This study aimed to evaluate the psychometric properties of the newly developed Social Isolation and Social Network (SISN) Scale among community-dwelling older adults in South Korea.

**Methods:**

A total of 350 community-dwelling older adults aged >65 years completed the SISN and Korean version of the Lubben Social Network Scale (LSNS-K). Psychometric properties were assessed using reliability analysis (internal consistency and test-retest), construct validity (confirmatory factor analysis), and diagnostic accuracy (receiver operating characteristic [ROC] curve analysis).

**Results:**

The SISN demonstrated excellent internal consistency (Cronbach’s α = 0.94) and test-retest reliability (intraclass correlation coefficient = 0.929, 95% confidence interval [CI]: 0.902–0.948). Confirmatory factor analysis supported the two-dimensional structure of social isolation (χ² (14) = 38.151, root mean square error of approximation [RMSEA] = 0.070, comparative fit index [CFI] = 0.983, Tucker–Lewis index [TLI] = 0.974) and social network (χ² (44) = 106.295, RMSEA = 0.064, CFI = 0.976, TLI = 0.964). The SISN showed a strong correlation with the LSNS-K (r = 0.785, p < .001). ROC curve analysis revealed gooddiscriminative ability (area under the curve = 0.900, 95% CI: 0.866–0.933), with an optimal cutoff score of 3.24 (sensitivity 81.8%, specificity 88.2%) for identifying social isolation risk.

**Conclusions:**

The SISN provided strong preliminary evidence of reliability and validity as a measure of social isolation and networks among older Korean adults. While these findings support its potential for research and clinical applications, the limited representativeness of the sample warrants cautious interpretation. Future studies with more diverse populations are recommended to strengthen generalizability.

## Introduction

Social isolation, defined as the absence of or limited access to social resources and relationships, has been extensively linked to negative health outcomes across psychological and physical domains [1–3]. Social isolation elevates the risk of mental health issues, such as depression, anxiety, and suicidal ideation [4], as well as physiological conditions, including cardiovascular disease and immune dysfunction [5]. Moreover, the growing reliance on digital interaction in contemporary society may have altered the nature of physical and social networks, potentially leading to emerging patterns ofsocial isolation [6,7]. Accordingly, there is an urgent need for tools capable of comprehensively evaluating social isolation and its associated networks to guide targeted interventions.

Social networks encapsulate individuals’ connections and the nature of these interactions, encompassing both quantitative and qualitative dimensions [8,9]. Robust social networks are consistently associated with improved mental and physical health outcomes [10–12]. Despite the well-established importance of social networks conceptual clarity on the distinctions and interrelations between social isolation and social networks remains insufficient in many studies [13,14]. While social isolation generally refers to an objective lack of social ties, social networks involve both structural aspects such as network size and density and functional aspects, including emotional support and perceived quality of relationships [13,15,16]. Addressing the multidimensionality of social connectedness, including emotional, structural, and functional layers, is thus essential [17]. To align with theoretical frameworks such as Berkman & Glass (2000), Cacioppo & Hawkley (2009), and the UCLA Loneliness Model, the SISN subscales are conceptually mapped as follows. The emotional layer captures perceived closeness and affective support, the structural layer measures network size, frequency of contact, and embeddedness, and the functional layer assesses the perceived availability of support and quality of interactions. The SISN incorporates two primary domains: Objective Isolation (7 items) and Social Network (12 items), designed to comprehensively represent these layers. This mapping ensures conceptual consistency and clarifies the measurement logic of each SISN subdomain [2,3].

In addition to these conceptual challenges existing tools such as the Lubben Social Network Scale (LSNS) and other brief instruments have been widely used [18]. However, they often emphasize quantitative metrics like the number of contacts or frequency of interactions while neglecting qualitative and culturally sensitive dimensions [19,20]. Furthermore, these scales may not sufficiently capture context-specific nuances, limiting their utility in diverse cultural settings such as Korea [21]. Therefore, there is a critical need to develop new tools that provide comprehensive, culturally adapted assessments that address these gaps [22].

The newly developed Social Isolation and Social Network (SISN) Scale aims to bridge these gaps by integrating both quantitative and qualitative aspects of social connectivity, emphasizing cultural relevance and conceptual depth. Specifically, the SISN considers emotional closeness, perceived support, and structural embeddedness to comprehensively profile social connectedness [23].

Tools assessing social isolation often rely on self-reported data or frequency-based interaction measures [24]. Although these methods are valuable, they lack sensitivity to the broader dynamic aspects of social connectivity, leading to potential limitations in reliability and construct validity [25]. Existing tools frequently fail to address critical diversity factors such as cultural or age-specific variations [24]. Developing a new tool that bridges these gaps and provides a multidimensional, culturally sensitive measurement of social isolation and network structure is fundamental for overcoming these challenges.

Reliability and validity are the two pillars of psychometric evaluation and form the foundation of standardized measurement tools [26]. Reliability, evaluated through internal consistency and test-retest methods, ensures measurement stability and internal coherenceof the tool over time [27]. Specifically, internal consistency measures whether individual items within the tool align cohesively to assess the intended constructs [28]. Conversely, validity gauges the extent to which a tool successfully measures the target theoretical framework. This study emphasized both reliability and validity by employing established psychometric methods to support clinical applicability and cross-cultural generalizability [29].

Receiver operating characteristic (ROC) curve analysis is a widely recognized statistical method for evaluating the diagnostic accuracy of measurement tools [30]. This technique assesses the balance between sensitivity and specificity, making it particularly useful for validating psychological and social assessments [31]. By implementing ROC curve analysis, the present study explored whether the newly designed tool could provide complementary value to traditional instruments such as the LSNS in detecting and distinguishing between varying levels of social isolation and network patterns [32]. This approach enhances understanding of the tool’s strengths and limitations in various cultural contexts, thus informing future adaptation and validation efforts.

The study of social isolation and connectedness requires a multidisciplinary perspective encompassing psychology, sociology, public health, and data science [33,34]. Effective tool development not only requires rigorous psychometric evaluation but also ensures relevance to real-world applications that can inform public health initiatives [35]. The present study aligns with the ongoing demand for holistic methodologies that address complex social phenomena using a coordinated cross-disciplinary framework [36].

This study sought to establish the reliability and validity of a newly developed social isolation and network profiling tool. Specifically, it evaluated psychometric reliability through internal consistency and test-retest analysis while validating diagnostic efficiency through ROC curve analysis. The ultimate goal was to provide a standardized, nuanced instrument for assessing social isolation and connectivity that has both clinical relevance and public health implications. This study addresses a critical gap in the existing toolkit for social evaluation and lays the groundwork for future interdisciplinary approaches to alleviate the adverse effects of social disconnection.

## Methods

### Participants

The participants in this study were community-dwelling older adults aged over 65 years residing in South Korea, who were cognitively intact and able to communicate fluently in Korean. No specific exclusion criteria were applied.

A non-probabilistic convenience sampling approach was employed for participant recruitment. In total, 350 participants were recruited from an online research company (www.embrain.com). The company had 1,428,252 research panels composed of respondents who had previously announced their intention to participate in surveys and provided personal information through a contract with the research company. Individuals who agreed to participate checked the consent tick box on the first page of the survey. Participants were selected based on availability and willingness to participate rather than through random selection from the target population. Eligible participants from the research panel who met the inclusion criteria (age ≥ 65 years, Korean fluency, cognitive intactness) were invited to participate via the online platform.

All participants’ capacity to provide informed consent was assessed through the inclusion criteria requiring cognitive intactness and fluency in Korean language communication. Participants demonstrated their cognitive capacity and understanding by successfully navigating the online survey platform independently, reading the study information, and completing the informed consent process autonomously. The consent process required participants to actively check the consent checkbox after reviewing the study details, confirming their voluntary participation and understanding of the research procedures.

Individuals who agreed to participate provided informed consent by checking the consent tick box on the first page of the survey. Data collection was conducted between February 14, 2025, and March 6, 2025. The Baekseok University Institutional Review Board (BUIRB-202501-HR-072) reviewed and approved this consent procedure and deemed it appropriate for the study population.

### Instruments

#### Social Isolation and Social Network Scale (SISN).

The SISN scale was developed through a rigorous two-phase process involving systematic literature review and expert consensus validation using a modified Delphi technique **[23]**

Phase 1: Literature-based Item Development: The initial item pool was generated through a comprehensive literature review based on established theoretical frameworks. The scale development was grounded in Berkman and Syme’s (1979) [37] social network theory, Cornwell and Waite’s (2009) [5] conceptual distinction between objective and subjective isolation, and Cacioppo and Hawkley’s (2009) [38] framework for perceived social isolation. This systematic process yielded 35 items organized into three conceptual domains: objective social isolation (7 items), subjective social isolation (10 items), and social network (15 items).

Phase 2: Expert Consensus Validation: A modified Delphi survey was conducted with 23 multidisciplinary experts (70% occupational therapists, 17% physical therapists, 9% nurses, 4% social workers) with over 5 years of clinical experience in Korea. Through two rounds of evaluation using Content Validity Ratio (CVR) analysis with a threshold of ≥0.37, three items were removed due to insufficient consensus and one new item regarding social network service (SNS) usage was added based on expert recommendations. All final items achieved CVR ≥ 0.37, with the overall CVR reaching 0.87, demonstrating high consensus (0.31) and convergence (0.87) [23].

Final Scale Structure: The final SISN consists of 19 items across two primary dimensions: Objective Isolation (7 items) and Social Network (12 items). The Objective Isolation domain includes items on frequency of leaving home, daily contact, SNS usage, and perceived disconnection. The Social Network domain measures family and friend network size, emotional closeness, availability of support, and quality of interactions. All items are rated on a five-point Likert scale ranging from 0 (“not at all”) to 4 (“very much”), with higher scores reflecting stronger social connectedness and more robust network integration. No reverse coding was required, and domain scores were calculated as item means. Thus, the observed subscale means (e.g., 0.791–1.746 for the Social Network domain) fall within the expected 0–4 scale range. (S1 File).

#### Lubben Social Network Scale – Korean version (LSNS-K).

The LSNS-K was used to examine the concurrent validity of the SISN. The LSNS was originally developed as a general measure of social ties to capture key features of older adults’ social networks. The Korean version was validated through a systematic translation and validation process. The LSNS-K consists of 10 self-administered items that measure three content areas: family and friend networks, interdependent social support, and living arrangements. The scale has demonstrated good psychometric properties in the Korean older adult population, with a Cronbach’s alpha of 0.75 and a four-week test-retest reliability coefficient of 0.78 (p < 0.0001) [39]. The LSNS-K was selected for the concurrent validity assessment because of its established validity and reliability in measuring social network characteristics among older Korean adults, making it an appropriate comparison measure for the newly developed SISN scale.

Table 1 demonstrates a comparative analysis of the SISN against commonly used social isolation and network assessment tools, highlighting the unique features and domains covered by each instrument. This comparison demonstrates how the SISN addresses gaps in existing tools by incorporating both objective and subjective dimensions of social connectedness, with particular attention to cultural relevance and emotional quality of relationships.

**Table 1:** Comparative features of social isolation and network assessment tools.

Assessment Domain	SISN	LSNS-K	UCLA Loneliness Scale-3
Objective Isolation	- Frequency of leaving home - Daily contact patterns - SNS usage frequency - Physical activity participation	Not assessed	Not assessed
Subjective/Emotional Quality	- Family network size (4 items) - Friend network composition (4 items) - Support network diversity (4 items	- Family ties (5 items) - Friend ties (5 items)	Not assessed
Network Structur	- Perceived closeness - Emotional support quality - Satisfaction with relationships - Sense of belonging	Limited assessment	- Feeling left out - Lack of companionship - Feeling isolated
Cultural Sensitivity	- Korean collectivist values - Multigenerational context - Digital integration (SNS) - Community embeddedness	Basic Korean translation	Limited cultural adaptation
Scoring Range	5-point Likert (0–4) “Not at all” to “Very much”	6-point scale (0–5)	4-point scale (1–4) “Never” to “Often”
Scoring Approach	Domain means Higher = better connectedness	Sum scores Higher = better networks	Sum scores Higher = more loneliness

*Note.* SISN = Social Isolation and Social Network Scale; LSNS-K = Korean version of Lubben Social Network Scale; SNS = Social Network Service. Scoring directions vary: higher SISN/LSNS-K scores indicate better connectedness, while higher UCLA scores indicate greater loneliness.

### Data analysis

First, both descriptive statistics and frequency analyses were used to analyze the demographic data. To verify the reliability of the SISN, Cronbach’s alpha was calculated for internal consistency. It is “Acceptable” when Cronbach’s alpha is between 0.70 and 0.80, “Good” when it is between 0.80 and 0.90, and “Excellent” when it is above 0.90 [40].

To verify the validity of the SISN, a Pearson’s correlation analysis was performed as the concurrent validity. For construct validity, a confirmatory factor analysis (CFA) was conducted to evaluate the goodness-of-fit of the model. The SISN constructs were treated as reflective measurement models, where observed items serve as indicators reflecting the underlying latent constructs of social isolation and social network.

Both a first-order two-factor model and a second-order factor model were examined to determine the optimal factor structure. The two-factor model was selected based on theoretical considerations from the scale development process and empirical evidence from the expert consensus validation. Model specification decisions were guided by modification indices, which were examined to identify potential areas for model improvement. However, modifications were only implemented if they were theoretically justifiable and did not compromise the conceptual integrity of the scale.

The goodness-of-fit indices used in this study were the Tucker–Lewis Index (TLI), Comparative Fit Index (CFI), and Root Mean Square Error of Approximation (RMSEA). When TLI or CFI is ≥ 0.90, the goodness-of-fit for the model is “Acceptable”. When the RMSEA is ≤ 0.08, the goodness-of-fit for the model is “Acceptable.” [41] Statistical significance of factor loadings was evaluated using z-values, with standardized factor loadings and their significance levels reported to assess the adequacy of individual items. Lastly, to calculate the optimal cutoff score that can discriminate between the normal and socially isolated groups, sensitivity, specificity, and predictive values were calculated using the ROC curve. The diagnostic accuracy of the SISN in distinguishing between normal older adults and older adults with social isolation risk was evaluated using ROC curve analysis, with the LSNS-K serving as the reference standard to define the criterion condition. Social isolation risk was defined as an LSNS-K score < 20.

The CFA was conducted using Mplus 8.0. Other statistical analyses were performed using the predictive analytics software Statistics for Windows, version 18.0.0 (SPSS, Chicago, IL, USA).

## Results

### Participants

Table 2 presents the demographic characteristics and descriptive statistics of the 350 participants. The mean age of participants was 68.95 years (SD = 3.98). Additionally, 227 (64.9%) participants had college degrees. The mean SISN was 3.32 (SD = 0.72) and the mean LSNS-K was 2.06 (SD = 0.73).

**Table 2:** Demographic characteristics and descriptive statistics of community-dwelling korean older adults (N = 350).

Variable	Category	N	(%)
Sex	Male	175	50.0
	Female	175	50.0
Age (years)	65–69	231	66.0
	70–79	109	31.1
	≥ 80	10	2.9
Educational Attainment	No Formal Education	1	0.3
	Elementary School	2	0.6
	Middle School	10	2.9
	High School	106	30.3
	College or University	227	64.9
	No Response	4	1.1

*Note.* M = 68.95 years, SD = 3.98 years for age. SISN total score: M = 3.32, SD = 0.72. LSNS-K total score: M = 2.06, SD = 0.73. SISN = Social Isolation and Social Network Scale; LSNS-K = Korean version of Lubben Social Network Scale.

### Reliability

The Cronbach’s alpha for the total SISN scale was 0.94, indicating excellent internal consistency (Table 3). While this high coefficient raises considerations about potential item redundancy, the two-dimensional factor structure confirmed through CFA and the distinct factor loadings (ranging from 0.521–0.917 for social isolation and 0.519–0.916 for social network) suggest that items contribute meaningfully to their respective constructs without excessive overlap. Additionally, the strong model fit indices and the theoretical distinctiveness of the two subscales support the appropriateness of the current item structure.

**Table 3:** Internal Consistency and test-retest reliability of the social isolation and social network scale (N = 350).

Scale	Items	Cronbach’s α	Composite Reliability	Test-retest ICC	95% CI
Total SISN Scale	19	0.94	.94	0.929	[0.902-0.948]
Social Isolation Subscale	7	0.92	.93	0.873	[0.825-0.908]
Social Network Subscale	12	0.94	.95	0.918	[0.887-0.941]

*Note.* Test-retest reliability assessed with n = 150 participants over a two-week interval. CI = confidence interval; ICC = intraclass correlation coefficient; SISN = Social Isolation and Social Network Scale. ***p < .001.

Specifically, Cronbach’s alpha was 0.92 for the social isolation subscale (7 items) and 0.94 for the social network subscale (12 items). Composite reliability was also calculated to provide a more robust estimate of internal consistency, yielding values of 0.93 for the social isolation subscale and 0.95 for the social network subscale, which were consistent with the Cronbach’s alpha values and further confirmed excellent internal consistency.

Test-retest reliability was assessed in 150 participants who completed the SISN twice over a two-week interval (Table 3). The intraclass correlation coefficient (ICC) for the total scale was 0.929 (95% confidence interval [CI]: 0.902–0.948, *p* < 0.001), indicating excellent temporal stability. The subscales also demonstrated strong test-retest reliability, with ICC values of 0.873 (95% CI: 0.825–0.908, *p* < 0.001) for the social isolation subscale (seven items) and 0.918 (95% CI: 0.887–0.941, *p* < 0.001) for the social network subscale (12 items).

### Construct validity

The construct validity of the SISN was evaluated via CFA using the Mplus software. Initial analysis compared the fit of a single-factor model against the hypothesized two-factor model, with the two-factor model demonstrating superior fit. A second-order factor model was also tested but did not provide significantly better fit than the first-order two-factor model, supporting the retention of the more parsimonious two-factor structure.

Analyses were conducted separately for the social isolation (7 items) and social network (12 items) subscales. For the social isolation subscale, the CFA results demonstrated good model fit: χ² (14) = 38.151, p < .001; RMSEA = 0.070 (90% CI: 0.044–0.097); CFI = 0.983; TLI = 0.974; SRMR = 0.028. All items showed significant factor loadings with z-values ranging from 9.17 to 10.99 (all p < .001) ranging from 0.790–0.917 (p < .001), with standardized regression weights (β) ranging from 0.271–0.838.

For the social network subscale, the CFA results also indicated good model fit: χ² (44) = 106.295, p < .001; RMSEA = 0.064 (90% CI: 0.048–0.079); CFI = 0.976; TLI = 0.964; SRMR = 0.033. All items demonstrated significant factor loadings with z-values ranging from 9.21 to 13.92 (all p < .001), confirming the hypothesized factor structure of the social network dimension. Examination of modification indices revealed no theoretically justified modifications that would substantially improve model fit, supporting the appropriateness of the final model specification.

Item analysis revealed that all items meaningfully contributed to their respective subscales. The mean scores for social isolation items ranged from 3.117–3.997, whereas those for social network items ranged from 0.791–1.746. All items were measured on a 5-point Likert scale. Item-total correlations were satisfactory, indicating good discriminant properties of the individual items. The results of the CFA support the two-dimensional structure of the SISN, confirming that the instrument effectively measured both social isolation and social network constructs as distinct but related dimensions of social connectedness (Table 4).

**Table 4:** Confirmatory factor analysis fit indices for the social isolation and social network scale (N = 350).

Subscale	Model fit indices				
	χ² (df, p)	RMSEA (90% CI)	CFI	TLI	SRMR
Social Isolation Subscale	38.151 (14, p < .001)	0.070 (0.044–0.097)	0.983	0.974	0.028
Social Network Subscale	106.295 (44, p < .001)	0.064 (0.048–0.079)	0.976	0.964	0.033

*Note.* CFI = comparative fit index; TLI = Tucker-Lewis index; RMSEA = root mean square error of approximation; SRMR = standardized root mean square residual; CI = confidence interval.

### ROC analysis

ROC analysis was performed to determine the cutoff score of the SISN that can identify older adults with social isolation risk using the LSNS-K as the reference standard. ROC analysis revealed that the SISN demonstrated good discriminative ability, with an area under the curve (AUC) of 0.900 (95% CI: 0.866–0.933), which was statistically significant (p < .001). This indicates good diagnostic accuracy for identifying social isolation risk as defined by the LSNS-K reference standard. This indicates that the SISN had an good discriminative validityfor identifying individuals at risk of social isolation.

**Fig 1:**
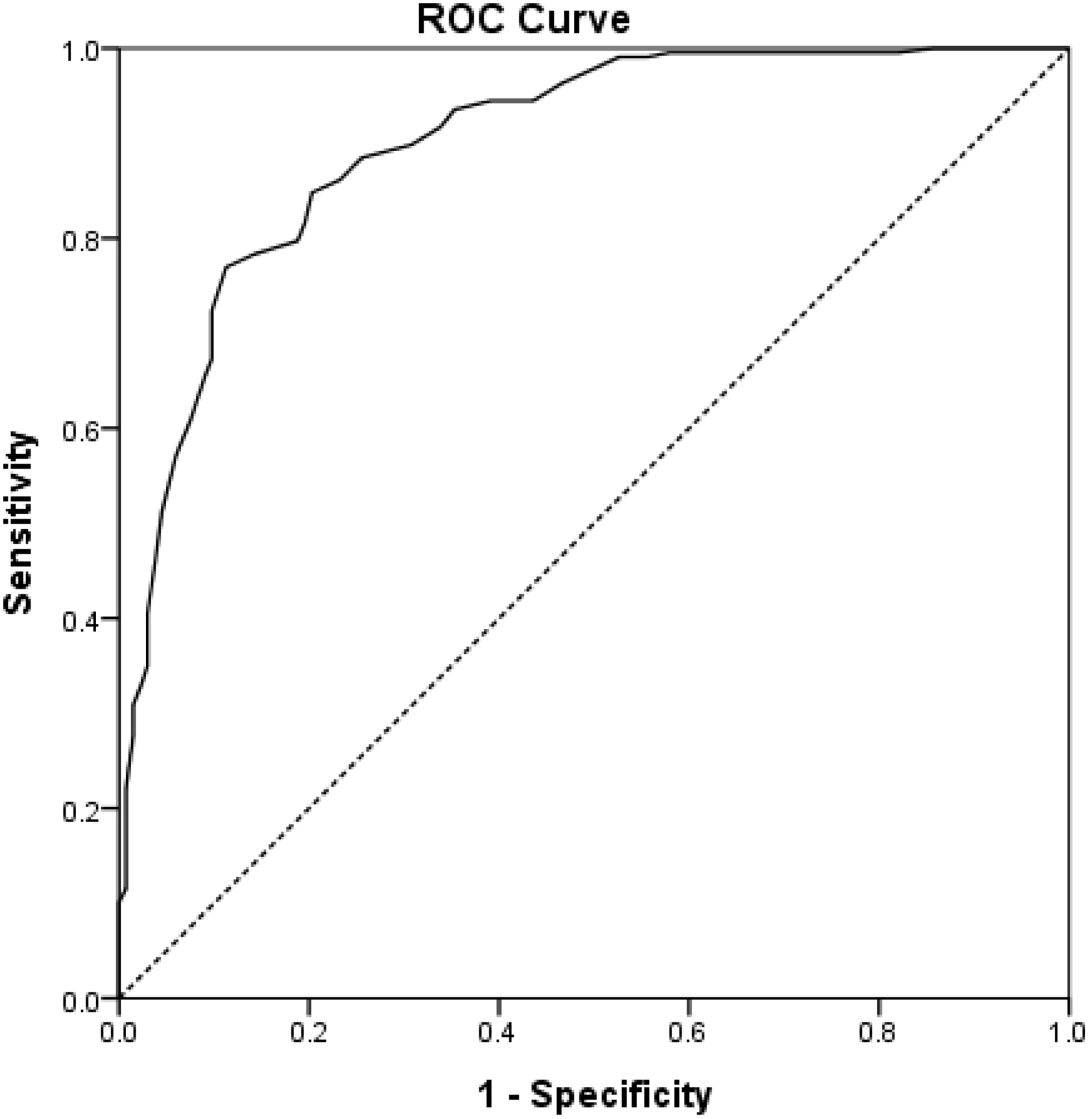
ROC curve for the SISN to investigate the optimal cut-off score.

Using the Youden index to maximize both sensitivity and specificity, the optimal cutoff score was determined to be 3.24. At this threshold, the SISN demonstrated 81.8% sensitivity and 88.2% specificity (Table 5, Fig 1).

**Table 5:** Diagnostic accuracy of the social isolation and social network scale for identifying social isolation risk.

Measure	AUC (95% CI)	Sensitivity (%)	Specificity (%)	Youden index	Cut-off score
SISN	0.900 (0.866–0.933)*	81.8	88.2	0.693	3.24

*Note.* Social isolation risk defined as LSNS-K score < 20. AUC = area under the curve; CI = confidence interval; SISN = Social Isolation and Social Network Scale; LSNS-K = Korean version of Lubben Social Network Scale. ***p < .001.

Correlation analysis between the SISN and LSNS-10 scores showed a strong positive correlation (r = 0.785, p < .001), further confirming that the SISN measures social isolation and social network constructs in a manner highly consistent with the established LSNS-10 measure. Based on the ROC analysis results, SISN mean scores < 3.24 indicated a high risk for social isolation, whereas scores ≥ 3.24 suggested a low risk for social isolation (good social connectedness).

## Discussion

This study evaluated the psychometric properties of the SISN Scale among community-dwelling older adults in South Korea. The results demonstrated preliminary psychometric evidence, supporting the utility of the scale as a robust assessment tool for measuring SISN dimensions. These findings are consistent with those of prior research and highlight the importance of capturing the multidimensional aspects of social connectedness in aging populations [42].

Notably, the SISN’s distinct yet interconnected subscales offer essential insights for clinical applications and public health interventions aimed at reducing social isolation and enhancing networks among older adults.

The reliability indices of the SISN were particularly strong, with a Cronbach’s alpha of 0.94 for the total scale and similarly high values for the subscales of social isolation and social networks (0.92 and 0.94, respectively). While these results suggest high internal consistency, the elevated Cronbach’s alpha may also indicate potential item redundancy.

Further analyses, such as inter-item correlations, item-total correlations, and item response theory (IRT) modeling, could help identify and refine overlapping items [40]. In particular, Future research should explore whether a reduced item set could maintain psychometric robustness while enhancing practical utility.

These findings are consistent with similar results from previous studies on social isolation scales that found high reliability in similar psychometric assessments [42]. However, contrasting evidence from a study by Shankar et al. [43] reported moderately lower reliability of tools assessing social isolation, suggesting that cultural or contextual factors may influence reliability outcomes. To address this, the revised discussion now clarifies that these cultural factors should not be assumed but rather examined through measurement invariance testing across diverse demographic and cultural subgroups [44].

This discrepancy may arise from the differing operational definitions of social isolation and diverging measurement practices across studies. Shankar et al. [43] highlighted the potential for overreliance on Western-centric measurement frameworks, which may not fully account for the collectivist cultural norms predominant in countries such as South Korea.

By contrast, the present study, conducted in a collectivist society, demonstrates how culturally sensitive adaptations of measurement tools can yield more robust and reliable outcomes. These comparisons underscore the need for cross-cultural measurement invariance testing and further scale refinement to ensure generalizability across different cultures [42].

Construct validity was well-supported by a CFA, with fit indices (RMSEA, CFI, and TLI) meeting or exceeding benchmarks. These outcomes reinforce the SISN’s hypothesized two-dimensional structure comprising the social isolation and social network subscales. This finding supports previous research suggesting that social connectedness encompasses distinct, yet interrelated constructs [5,45].

However, Western studies have reported greater overlap between these domains [46,47], raising questions about whether collectivist cultural contexts, such as South Korea, facilitate clearer separation. This nuance has been emphasized to avoid speculative cultural interpretations and to propose future cross-cultural invariance testing as a research priority.For example, Cornwell and Waite [45] argued that the boundaries between these constructs are blurred when assessing populations with high levels of interdependence, such as older adults who live in communal settings. This discrepancy may be attributed to contextual differences in social organizations. Older adults in South Korea often rely on multigenerational households to establish community networks, potentially contributing to a clearer demarcation between social isolation and networks. Further comparative analyses across diverse living arrangements are required to clarify these differences.

Although CFA supports structural validity, complementary psychometric methods such as Rasch analysis or differential item functioning (DIF) analysis can further examine item fairness and functioning across demographic subgroups, including sex, socioeconomic status, and educational levels [48]. These advanced approaches will help enhance the scale’s applicability and fairness.

The SISN demonstrated high diagnostic accuracy in identifying older adults at risk of social isolation (AUC = 0.900). While this strong discriminatory ability aligns with prior studies [21], other research reported lower accuracy in cross-cultural contexts due to diverse social norms [49]. The high AUC in this study may reflect contextual relevance to South Korean older adults, where social roles emphasize familial and close social bonds. However, reliance on LSNS-K as a reference standard in ROC analysis could introduce circularity, as both scales target similar constructs [18,23]. This limitation should be addressed in future comparative validation studies using more independent criterion measures.

Despite its contributions, this study has some limitations. First, the study sample was limited to older adults in South Korea, which may restrict the generalizability of the findings. Given that the cultural norms related to social connectedness vary across countries and regions, the interpretation of “support” or “network” may differ substantially in rural or migrant populations, where resources and social structures are less urban-centric. This context-specific variability highlights the need for further validation studies in diverse geographic and socio-cultural settings are required to broaden the applicability of SISN. Coyle and Dugan (2012) highlighted the potential inability of culturally neutral tools to capture subtle differences in social relationships and emphasized the necessity of developing tools that account for cultural contexts [50].

Second, Second, convenience sampling via an online research panel limits sample representativeness and generalizability. Voluntary panel participation likely overrepresented older adults with greater social engagement or online experience, while underrepresenting those with limited digital access or higher social isolation. Furthermore, a very high proportion of respondents (95%) had at least a high school education, suggesting that the scale was tested largely in groups with relatively high social literacy and survey familiarity. These sampling biases constitute a critical limitation and may affect the reliability and construct validity analyses of the SISN, particularly for populations with lower educational attainment.

Third, this study employed a cross-sectional design, which prevented the assessment of changes in social connectedness over time. Although confirming the temporal stability of the SISN is a significant achievement, future research should incorporate longitudinal designs to evaluate how sensitively the scale responds to changes in the social environment. For instance, assessing whether a SISN adequately detects network changes following intervention programs is a critical task for future research [42].

Finally, it is important to further refine the assessment of item-level performance through advanced psychometric approaches such as Rasch modeling or Differential Item Functioning analysis. These techniques would allow for the evaluation of the fairness of the scale across demographic subgroups, including sex, socioeconomic status, and education level, thereby strengthening the applicability of the SISN to diverse populations.

## Conclusion

In conclusion, the SISN is a preliminarily validated and culturally informedtool for assessing social isolation and social networks among older South Korean adults. The findings of this study contribute to the growing literature on social isolation assessment while highlighting the need for culturally sensitive adaptations and longitudinal research. Despite certain limitations, the evidence of reliability, construct validity, and classification accuracyfor the SISN underscores its potential utility in both research and practice. Future research should include cross-cultural measurement invariance testing, alternative validation benchmarks, and longitudinal studies to strengthen generalizability and ecological validity. These steps will enhance the scale’s applicability to diverse aging populations and support broader implementation in clinical and community settings.

## Supporting information

S1 File SISN assessment. (PDF)
