# Supplementary material for: Psychometric properties of the social isolation and social network scale in community-dwelling older adults: Construct validity, reliability, and sensitivity
Source: PLoS One. 2025 Dec 11;20(12):e0338522. doi: 10.1371/journal.pone.0338522 (PMC12697958; doi:10.1371/journal.pone.0338522)
Supplement: S1 File — (PDF) [file pone.0338522.s001.pdf]

Social Isolation and Social Network (SISN)

I. Objective isolation

The following questions are about your objective isolation. Please mark 'v' in the appropriate box.

1. Please indicate how many times you go out of the house on average per day.

☐ Never

☐ Once

☐ 2-3 times

☐ 4-5 times

☐ 6 or more times

2. How many people call you or ask how you are every day?

☐ None

☐ 1 person

☐ 2 people

☐ 3-4 people

☐ 5 or more people

3. Are you currently communicating through Social Network Sites?

☐ Never

☐ Rarely

☐ Sometimes

☐ Often

☐ Very often

4. Over the past month, I've really missed my close friend.

☐ Strongly agree

☐ Agree

☐ Neutral

☐ Disagree

☐ Strongly disagree

5. Over the past month, I have felt a general sense of emptiness

☐ Strongly agree

☐ Agree

☐ Neutral

☐ Disagree

☐ Strongly disagree

6. Over the past month, I've felt like my circle of friends and acquaintances was too limited.

☐ Strongly agree

☐ Agree

☐ Neutral

☐ Disagree

☐ Strongly disagree

7. Over the past month, I have felt separated from others.

☐ Strongly agree

☐ Agree

☐ Neutral

☐ Disagree

☐ Strongly disagree

II. Social network

The following questions are about your social network. Please mark 'v' in the appropriate box.

1. How many family members and relatives do you see or hear from more than once a month?

☐ None

☐ 1 person

☐ 2 people

☐ 3-4 people

☐ 5 or more people

2. How many of your family and relatives do you feel comfortable enough to talk to about personal matters?

☐ None

☐ 1 person

☐ 2 people

☐ 3-4 people

☐ 5 or more people

3. How many family members and relatives do you feel close enough to ask for help?

☐ None

☐ 1 person

☐ 2 people

☐ 3-4 people

☐ 5 or more people

4. Is there always someone you can talk to about everyday problems?

☐ None

☐ 1 person

☐ 2 people

☐ 3-4 people

☐ 5 or more people

5. Are there a lot of people you can turn to when you have a problem?

☐ None

☐ 1 person

☐ 2 people

☐ 3-4 people

☐ 5 or more people

6. Are there many people I can completely trust?

☐ None

☐ 1 person

☐ 2 people

☐ 3-4 people

☐ 5 or more people

7. Do I have friends I can call whenever I need them?

☐ None

☐ 1 person

☐ 2 people

☐ 3-4 people

☐ 5 or more people

8. How many friends do you see or hear from more than once a month?

☐ None

☐ 1 person

☐ 2 people

☐ 3-4 people

☐ 5 or more people

9. How often do you see or hear from the friends you have the most contact with?

☐ Once a year

☐ Once every 6 months

☐ Once a month

☐ 2-3 times a week

☐ Once a day

10. How many friends do you have with whom you feel comfortable enough to talk about personal matters?

☐ None

☐ 1 person

☐ 2 people

☐ 3-4 people

☐ 5 or more people

11. How many friends do you feel close enough to ask for help?

☐ None

☐ 1 person

☐ 2 people

☐ 3-4 people

☐ 5 or more people

12. When one of your friends has to make an important decision, how often does he talk about it with you?

☐ Almost never

☐ Occasionally (1-2 times a week)

☐ Sometimes

☐ Often (5-6 times a week)

☐ Always
